# Detection and identification of tea leaf diseases based on AX-RetinaNet

**DOI:** 10.1038/s41598-022-06181-z

**Published:** 2022-02-09

**Authors:** Wenxia Bao, Tao Fan, Gensheng Hu, Dong Liang, Haidong Li

**Affiliations:** National Engineering Research Center for Agro-Ecological Big Data Analysis and Application, Anhui University, Hefei, Anhui China

**Keywords:** Image processing, Machine learning, Plant sciences

## Abstract

The accurate detection and identification of tea leaf diseases are conducive to its precise prevention and control. Convolutional neural network (CNN) can automatically extract the features of diseased tea leaves in the images. However, tea leaf images taken in natural environments have problems, such as complex backgrounds, dense leaves, and large-scale changes. The existing CNNs have low accuracy in detecting and identifying tea leaf diseases. This study proposes an improved RetinaNet target detection and identification network, AX-RetinaNet, which is used for the automatic detection and identification of tea leaf diseases in natural scene images. AX-RetinaNet uses an improved multiscale feature fusion module of the X-module and adds a channel attention module, Attention. The feature fusion module of the X-module obtains feature maps with rich information through multiple fusions of multi-scale features. The attention module assigns a network adaptively optimized weight to each feature map channel so that the network can select more effective features and reduce the interference of redundant features. This study also uses data augmentation methods to solve the problem of insufficient samples. Experimental results show the detection and identification accuracy of AX-RetinaNet for tea leaf diseases in natural scene images is better than the existing target detection and identification networks, such as SSD, RetinaNet, YOLO-v3, YOLO-v4, Centernet, M2det, and EfficientNet. The AX-RetinaNet detection and identification results indicated the mAP value of 93.83% and the F1-score value of 0.954. Compared with the original network, the mAP value, recall value, and identification accuracy increased by nearly 4%, by 4%, and by nearly 1.5%, respectively.

## Introduction

Tea is an important economic crop. It contains a variety of effective ingredients required by the human body, has medical and health care functions, and is quite effective in enhancing human immunity. Planting tea is an important way for tea farmers to make their fortunes. Currently, China’s tea planting area and output are the highest in the world. However, because of the effects of many diseases, such as tea algae leaf spot (TALS), tea bud blight (TBB), tea white scab (TWS), and tea leaf blight (TLB), the annual tea production has been reduced by as much as 20%^1^. Tea leaf diseases can also reduce the quality of tea and cause serious economic losses to tea farmers. Accurate detection and identification of tea leaf diseases and timely prevention and control measures are of great significance to reduce the loss of tea production, improve the quality of tea, and increase the income of tea farmers.

At present, the diagnosis of tea leaf diseases relies on the manual method. Most tea trees grow in rugged mountainous areas. Thus, it is time-consuming and costly for experts to go to the tea garden for diagnosis. However, results are largely subjective when famers rely on their own experience to distinguish the types of tea diseases. With the development of computer technology, the use of machine learning and image processing technology to achieve automatic detection and identification of crop diseases has become an important method for the automatic diagnosis of crop diseases^2,3^. Chaudhary et al. presented an improved Random Forest Classifier approach for multi-class disease classification of groundnut diseases. This approach combines the advantages of the Random Forest machine learning algorithm, an attribute evaluator method, and an instance filter method^4^. Hossain et al. developed an image processing system that can analyze 11 features of tea leaf diseases and used a support vector machine classifier to identify and classify the two most common tea leaf diseases in Bangladesh from healthy leaves, namely brown blight disease and algal leaf disease^5^. Sun et al. combined Simple Linear Iterative Cluster (SLIC) and Support Vector Machine (SVM) to improve the extraction of the saliency map of tea leaf diseases in complex backgrounds^6^. Hu et al. propose an estimation method for tea leaf blight severity in natural scene images. The method segmented disease spot regions from the images of tea leaf blight leaves through an SVM classifier to calculate the Initial Disease Severity (IDS) index. The IDS index, color features, and texture features are inputted into the metric learning model to estimate disease severity^7^. Tetila et al. used six classical machine learning methods to identify diseased soybean leaves in the images taken by an Unmanned Aerial Vehicle (UAV) at different heights and verified the effect of color and texture attributes based on the identification rate^8^. When classic machine learning methods, such as random forest and support vector machine, are used to diagnose plant diseases, extracting the disease features manually is necessary. The extracted features are not necessarily the disease’s essential features, which would considerably affect the accuracy of the disease diagnosis.

In recent years, deep learning, especially Convolutional Neural Networks (CNN), has been applied to crop pests and disease identification^9,10^. Compared with classical machine learning methods, deep learning methods can automatically extract crop disease features and have higher accuracy in identifying plant diseases. Jiang et al. combined deep learning and SVM methods to identify four rice diseases, among which CNN was used to extract the features of diseased rice leaves in the images, and the SVM was applied to classify and predict the specific disease^11^. Hu et al. proposed a low shot learning method that uses SVM to segment the disease spots in the images of diseased tea leaves to eliminate background interference, and used an improved conditional deep convolutional generative adversarial networks(C-DCGAN) to solve the problem of insufficient samples^12^. Xiong et al. presented an identification method for cash crop diseases using automatic image segmentation and deep learning with the expanded dataset. The idea of image segmentation was integrated to solve the problem of the insufficient generalization ability of CNNs in practical applications^13^. Tetila et al. evaluated the results of five deep learning methods using different fine-tuning and transfer learning strategies to classify soybean pests, and pointed out that the fine-tuned deep learning model can achieve better classification accuracy^14^. Krisnandi et al. concatenated GoogleNet, Xception, and Inception-ResNet-v2 for disease identification of tea plants, and confirmed the effectiveness of concatenated CNN in identifying tea plant diseases^15^. Hu et al. added a multiscale feature extraction module to the CIFAR10-quick model to identify tea leaf diseases, adopted depth-wise separable convolution to reduce the number of model parameters, and obtained good average identification accuracy^1^. Zhao et al. presented a Multi-Level Feature Pyramid Network (MLFPN) for detecting objects of different scales and a detector called M2Det was designed by integrating MLFPN into the architecture of SSD^16^. Although the abovementioned methods have achieved good results for the processing of crop diseases, they only involve a single task, namely, crop disease image identification or classification. The multi-task problems of detection and identification are not involved, thereby greatly limiting the application of these methods.

Faster R-CNN, a two-stage target detection network, uses a region proposal network (RPN) to detect the region of interest in the image and then uses a classifier to categorize the proposal box to achieve target detection and recognition^17,18^. Hu et al. used the Retinex algorithm to enhance the original image and reduce the influence of light variation and shadow, and employed an improved Faster R-CNN to detect tea leaf blight, and a VGG16 to estimate the severity of the disease^19^. However, the Faster R-CNN has slow detection speed, and has difficulty obtaining real-time results for high-resolution images. Tiwari et al. presented a dense convolutional neural network approach for plant disease detection and classification from leaf images captured in various resolutions. Images have several inter-class and intra-class variations with complex conditions that have been addressed in this dense neural network^20^. Redmon et al. proposed the You Only Look Once (YOLO) series of algorithms, which unify target classification and positioning into a regression problem and improve the speed of target detection^21–23^. Tian et al. used the improved YOLO-v3 for apple detection in three different growth periods in the orchard, and realized real-time detection of apples in the images while solving the problems of occlusion and overlap of leaves and branches^24^. Kang et al. developed a multi-task network DaSNeT-v2 for orchard visual sensing, which can perform detection and instance segmentation on fruits, and semantic segmentation on branches^25^. Yu et al. proposed a strawberry fruit detection algorithm based on Mask R-CNN, which overcomes the problems of poor universality and poor robustness of traditional machine vision in non-structural environments and offers a reference for the precise operation of harvesting robots^26^. Su et al. used a deep neural network to detect and segment the inter-row ryegrass in a wheat farm in real-time. The proposed method introduced two subnets to treat inter-row and intra-row pixels differently and provided corrections to preliminary segmentation results^27^. Lin et al. designed and trained a dense detector RetinaNet through the Focal loss function^28^. Focal loss is used to solve the problems of imbalance between positive and negative samples in the training process and the challenging classification of difficult samples so that the one-stage RetinaNet target detection network achieves the performance of two-stage target detection. RetinaNet does not need RPN because it directly regresses all the targets in the image to realize the detection of the target, which has high detection and identification accuracy and fast speed. Selvaraj et al. used UAV-RGB aerial images to develop a mixed-model system that combines RetinaNet and a custom classifier for simultaneous localization and disease classification of bananas, providing decision support for the prevention and control of major diseases of African bananas^29^.

The above deep neural networks have low accuracy in detecting and identifying tea leaf diseases in natural scene images because of the complex backgrounds, dense leaves, and large-scale changes of tea images taken in the natural environment. This study is based on the improved RetinaNet, namely AX-RetinaNet, to detect and identify tea diseases in images taken in the natural environment. AX-RetinaNet uses an improved multiscale feature fusion module X-module and adds a channel Attention module to enhance the detection and identification performance of the network^30^. The feature fusion module X-module obtains rich semantic information through multiple fusions of multi-scale features. The channel Attention module assigns the weight of the network adaptive optimization to each feature map channel so that the network can select more effective features and reduce the interference of irrelevant information.

The main contributions of this study are as follows:

1. An improved RetinaNet target detection model, AX-RetinaNet, is proposed for the automatic detection and identification of tea leaf diseases in natural scene images.
2. AX-RetinaNet uses an improved multiscale feature fusion module X-module to fuse the multiscale features of tea leaf diseases and obtain features of the diseases with rich semantic information.
3. AX-RetinaNet adds a channel Attention module, which adds adaptively optimized weights to each feature map channel to ensure that the network pays attention to useful feature information and reduces the interference of redundant information.
4. The data augmentation method is used to expand the number of training images to solve the problem of insufficient samples and improve the network detection and identification effect.
5. The method used in this study provides a basis for the automatic prevention and control of tea leaf diseases and is conducive to the rational use of pesticides.

The rest of the paper is organized as follows. In section “Materials and methods”, data acquisition, data augmentation, and annotation, improved RetinaNet, and algorithm steps and processes are included. In section “Experimental results and analysis”, experiments are carried out and the results of the experiments are discussed. Finally, section “Conclusion” provides the summary of this study.

## Materials and methods

### Data acquisition

The methods of experimental research and field research in this study are carried out according to relevant guidelines/regulations/legislation. During the research period, only the pictures of diseased leaves of tea were collected, and there are no other kinds of collection or sampling methods of other plants. The data are images of diseased tea leaves captured in the natural environment. The locations are Tianjingshan Tea Garden in the south of Hefei City, Anhui Province, China, and the Sanyuan Tea Garden in Wanzhi District, Wuhu City, Anhui Province, China. Tianjingshan Tea Garden is 40 m above sea level, 31°14′3″ north latitude and 117°36′16″ east longitude, and the acquisition time is April 6, 2019, and October 6, 2019, respectively. Sanyuan Tea Garden is located 20 m above sea level, 31°6′48″ north latitude, 118°36′29″ east longitude, and the acquisition time are June 26, 2020. The images of tea algae leaf spot and tea leaf blight were from Tianjingshan Tea Garden, whereas those of tea bud blight and tea white scab were from Sanyuan Tea Garden. It was sunny and well light at the time of the shooting. The images were captured using a Canon EOS 80D camera and a Sony DSC-W55 camera. The image resolution of the Canon EOS 80D camera is 6000 × 4000 pixels, the image resolution of the Sony DSC-W55 camera is 3072 × 2304 pixels. The cameras are placed 0.4 m above the tea tree canopy. Among the images of diseased tea leaves captured in the natural environments, 700 images of four types of tea leaf diseases were selected to construct a dataset, including 175 images of TALS, 175 images of TBB, 175 images of TWS, and 175 images of TLB.

**Figure 1:**
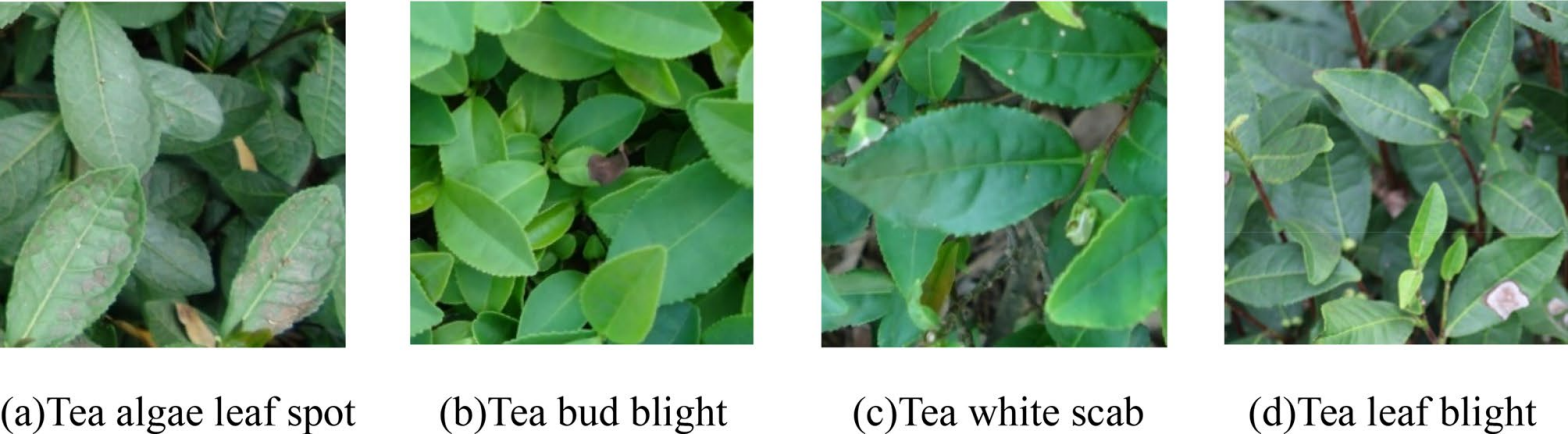
Examples of captured tea images of four tea leaf’s diseases.

The training set and test set were constructed according to the ratio of 4:1 for images of each type of tea leaf disease, that is, 140 images of each type of tea leaf disease were randomly selected to construct the training set, and 140 images of the remaining 35 images of each type of tea leaf disease were selected to construct the test set. Figure 1 shows the examples of captured tea images of these four tea leaf diseases.

### Image augmentation and annotation

Most tea plants grow in rugged mountainous areas, and different temperatures, seasons, and soils will cause the occurrence and spread of different tea leaf diseases. Because it is very difficult to collect tea images of different disease types, the number of collected images of tea leaves with different diseases is insufficient. A small number of training images will cause overfitting of the deep learning network and reduce the accuracy of the network in detecting and identifying tea leaf diseases. Hence, this study uses the image augmentation method to expand the number of tea images to solve the problem of insufficient training images, improve the generalization of the network, and prevent the network from overfitting.

**Figure 2:**
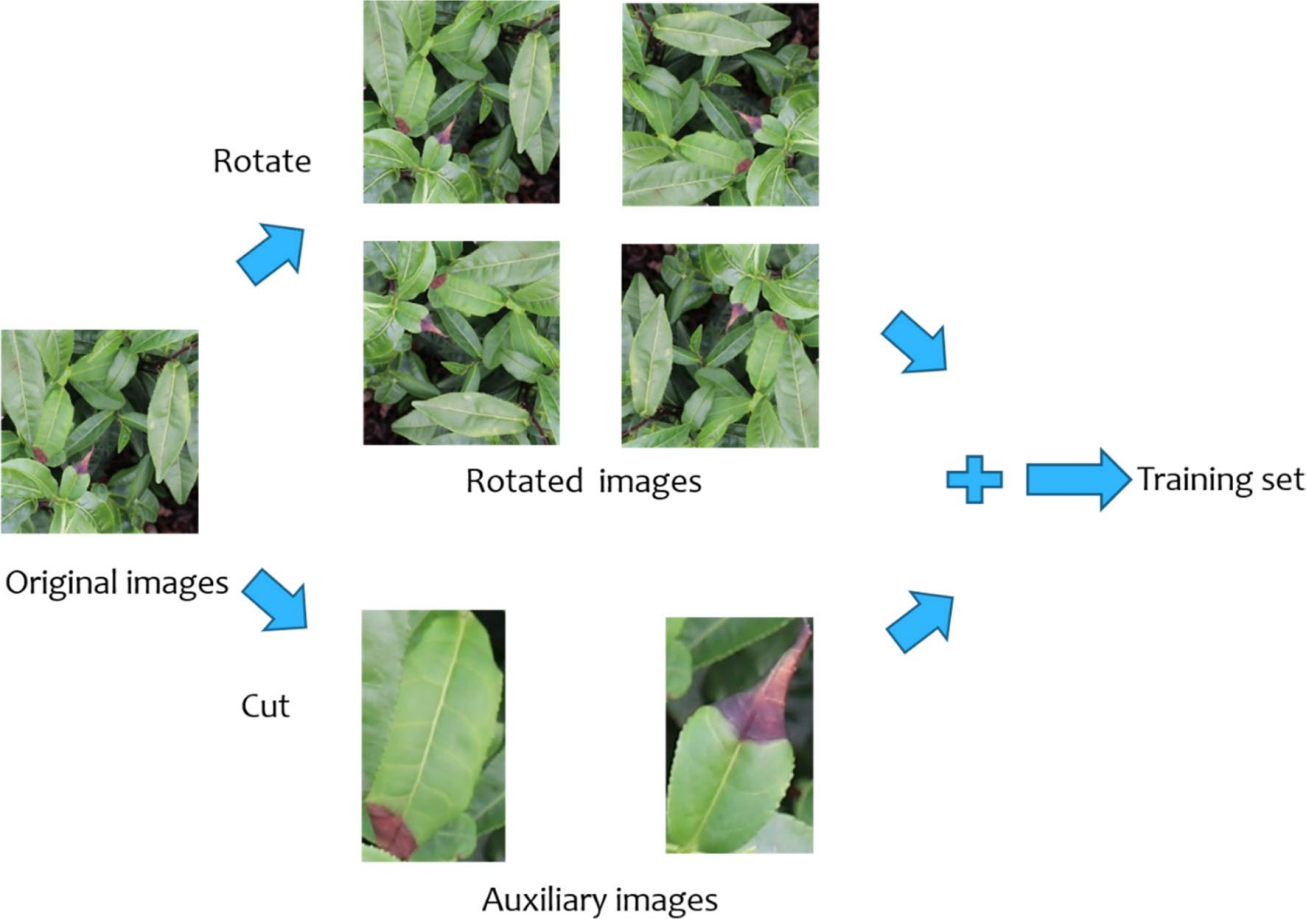
Schematic diagram of image augmentation method.

Figure 2 shows the schematic diagram of the image augmentation method used in this study. The target regions (the annotated regions) in the original images are cut as auxiliary images, and at the same time, the original images are rotated at four angles (90°, 180°, 270°, and 360°) to obtain the rotated images. The auxiliary images and the rotated images are merged to construct an augmented training image dataset. Compared with the rotation augmentation method, the proposed augmentation method adds the auxiliary images cut from the annotated regions in the original images, which not only expands the number of training images but also increases the diversity of the images, thereby allowing the network to focus more on the annotated regions during the training process, that is, the network is more sensitive to the annotated regions to improve detection and identification accuracy.

**Figure 3:**
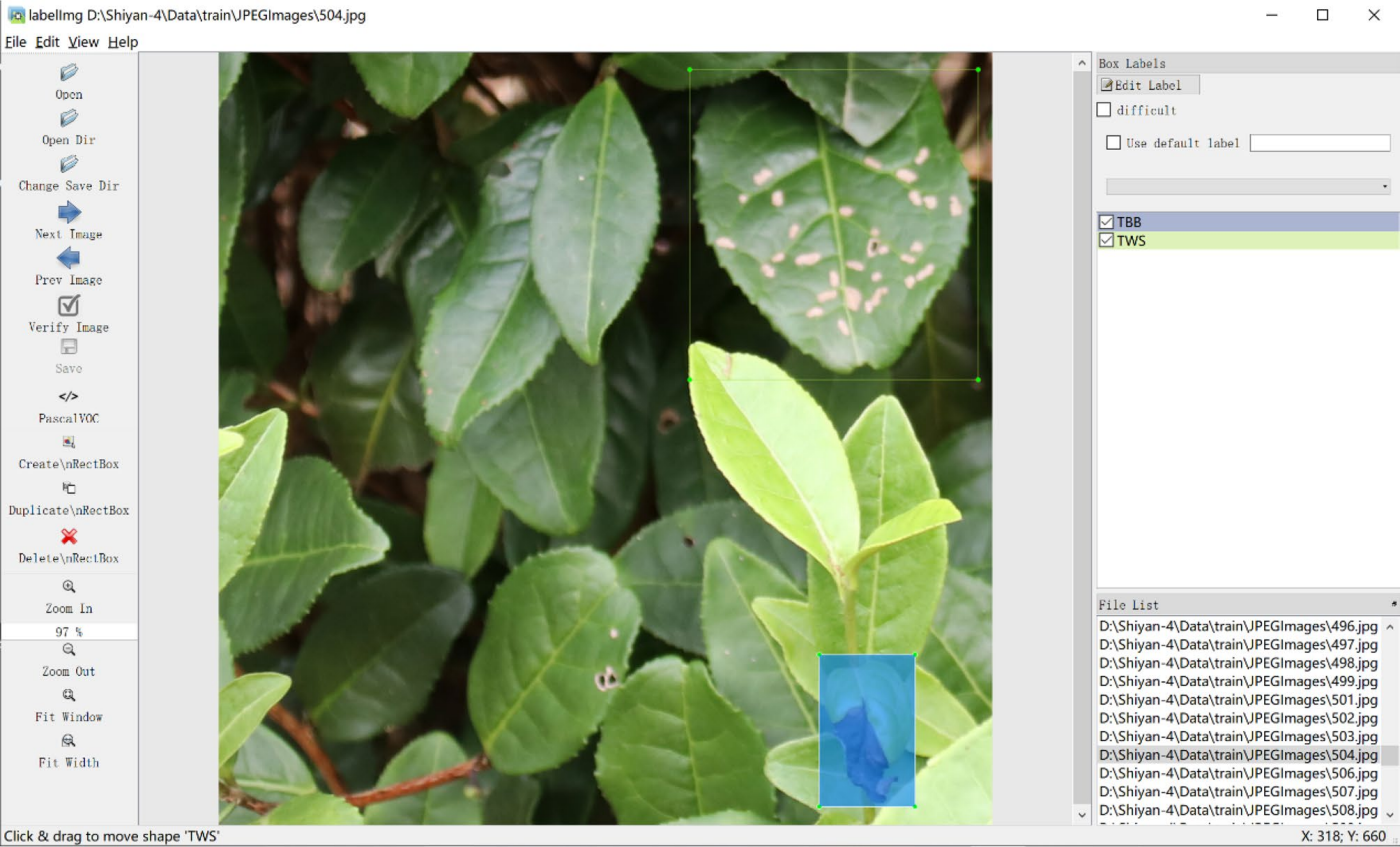
Using LabelImg tool to annotate tea leaf diseases and their locations.

In this study, the LabelImg tool was used to manually annotate the tea leaf diseases in the images, draw the bounding box and label the image with the disease type. Figure 3 shows an example of the use of the LabelImg tool to annotate tea leaf diseases and their locations. Because the tea disease images are captured in natural environments, some images are relatively blurry, and some of the diseased leaves in the images are occluded. Therefore, unclear diseased leaves and diseased leaves with an occlusion region greater than 85% are not annotated, diseased leaves that occupy a relatively small region at the edge of the images are not annotated, and other diseased leaves are annotated with their locations and disease types.

### Improved RetinaNet

#### RetinaNet

**Figure 4:**
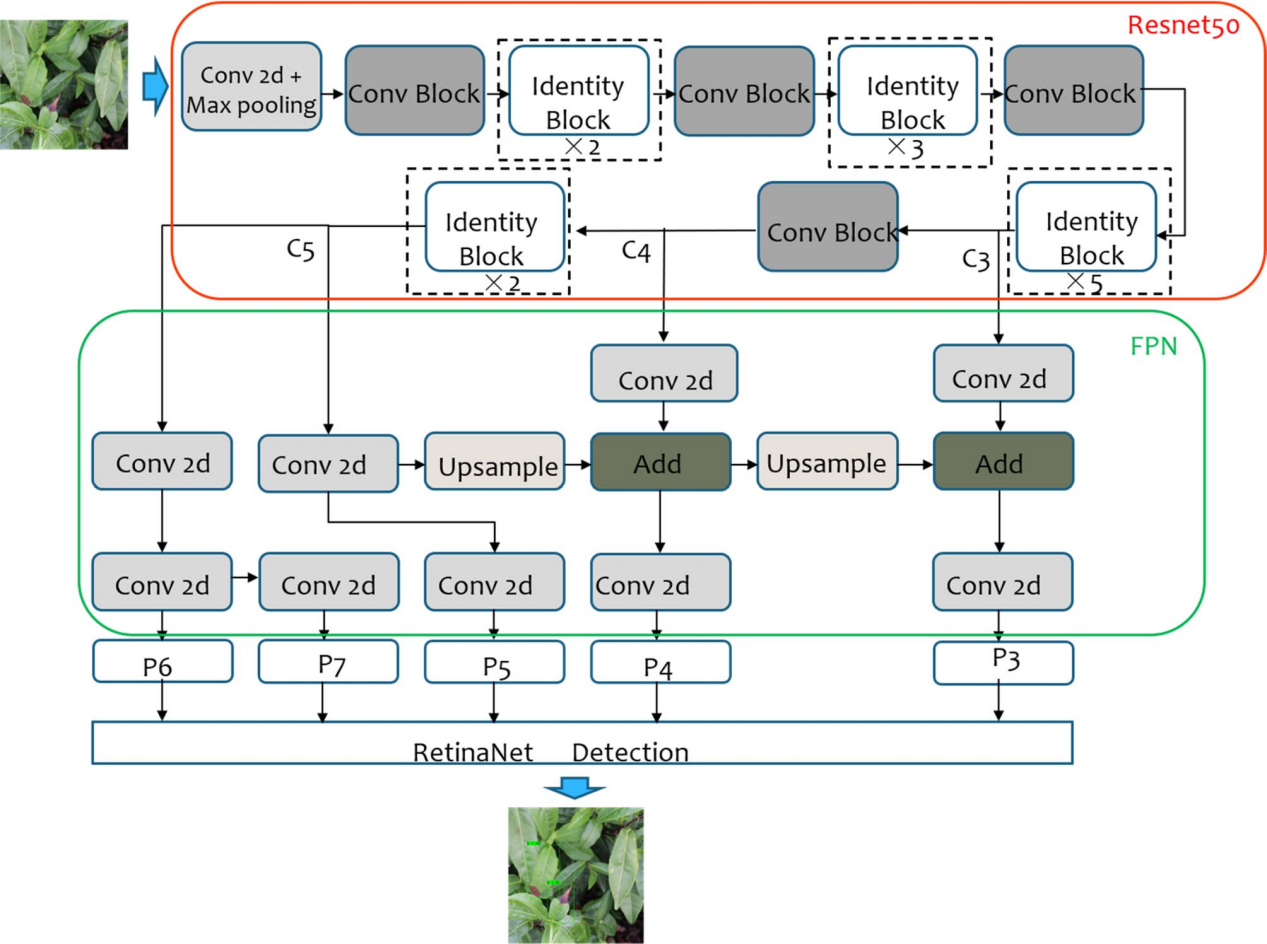
Structure of RetinaNet network.

The structure of RetinaNet is shown in Fig. 4. RetinaNet uses Resnet as the backbone network to extract the feature information of the target in the image. The features of the C3, C4, and C5 layers extracted by the backbone network pass through the Feature Pyramid Network (FPN) to obtain five effective feature layers, namely P3, P4, P5, P6, and P7. The five effective feature layers realize the detection and identification of the target through the regression and detection network constructed by the Fully Convolutional Network.

**Figure 5:**
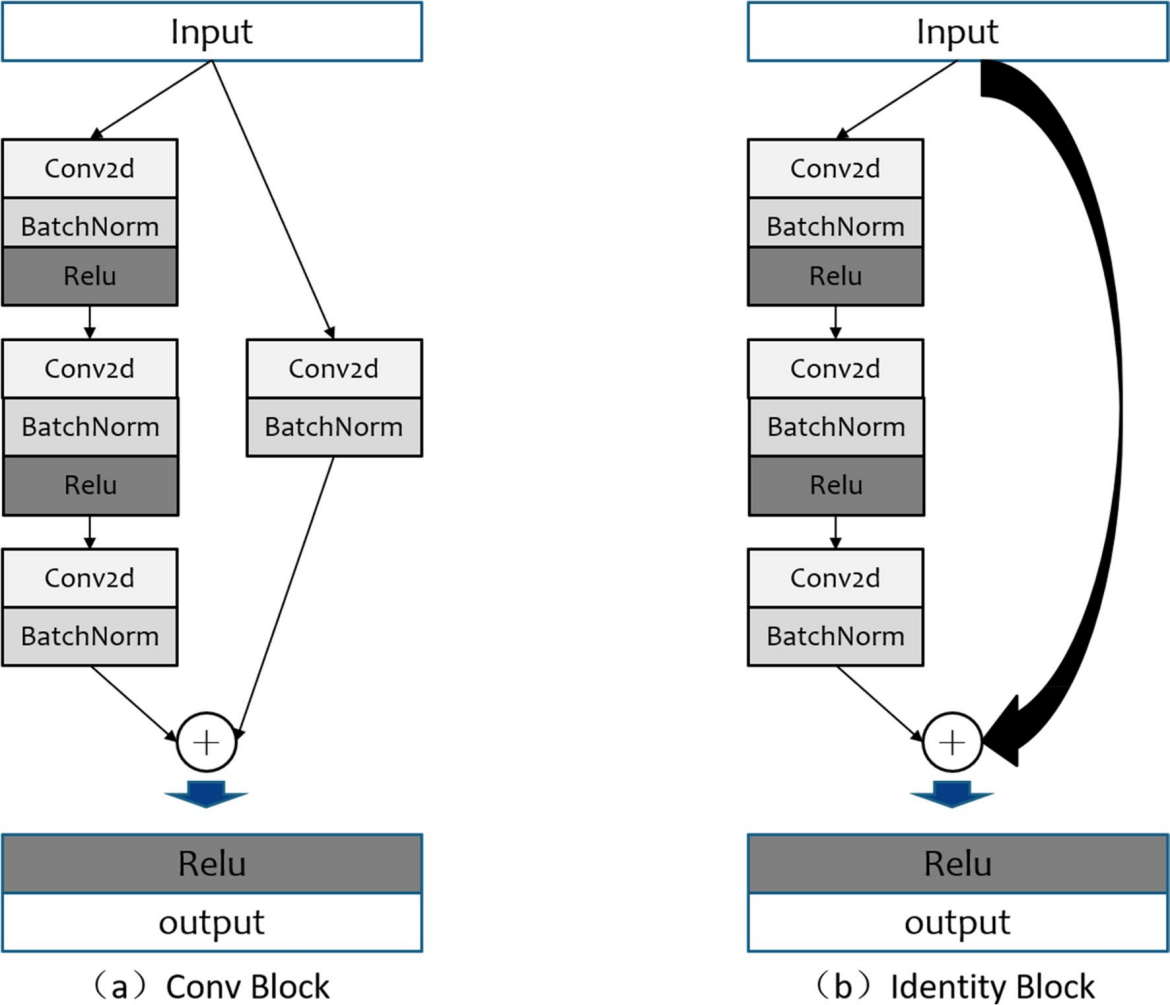
Two residual blocks in Resnet-50.

The backbone feature extraction network of RetinaNet can be Resnet-50 or Resnet-101^31^. In this study, Resnet-50 with fewer parameters is used as the backbone feature extraction network. In Resnet-50, the residual block is used to directly fuse the outputs of the shallow and deep layers of the network to overcome the problems of gradient dispersion and network degradation caused by the excessive depth of the network. The two residual blocks of Conv block and Identity block in Resnet-50 are shown in Fig. 5.

The FPN in RetinaNet solves the shortcomings of the classical target detection networks in dealing with the scale change of the target. Because small-scale targets have less pixel information, they are easily lost during the downsampling process. Thus, to deal with the problem of target detection with obvious scale differences, classical methods use image pyramids to extract multi-scale features, which involves considerable calculations. On the premise lessening the required calculation, FPN combines low-resolution feature maps with strong semantic information and high-resolution feature maps with weak semantic information but rich spatial information to obtain multi-scale feature information of the target and solve the problem of scale change of the target.

RetinaNet uses focal loss, in which the weight factor is used to adjust the ratio of positive samples to negative samples to solve the imbalance between the positive and negative samples in the training process and the difficult classification problem of difficult samples.

#### AX-RetinaNet

Although RetinaNet achieved better results compared with the existing classical networks, it has difficulty accurately detecting and identifying the tea leaf diseases in the images because of the complex backgrounds, dense leaves, and large-scale changes of images of diseased tea leaves taken in the natural environment. In this study, RetinaNet was improved and AX-RetinaNet was designed.

**Figure 6:**
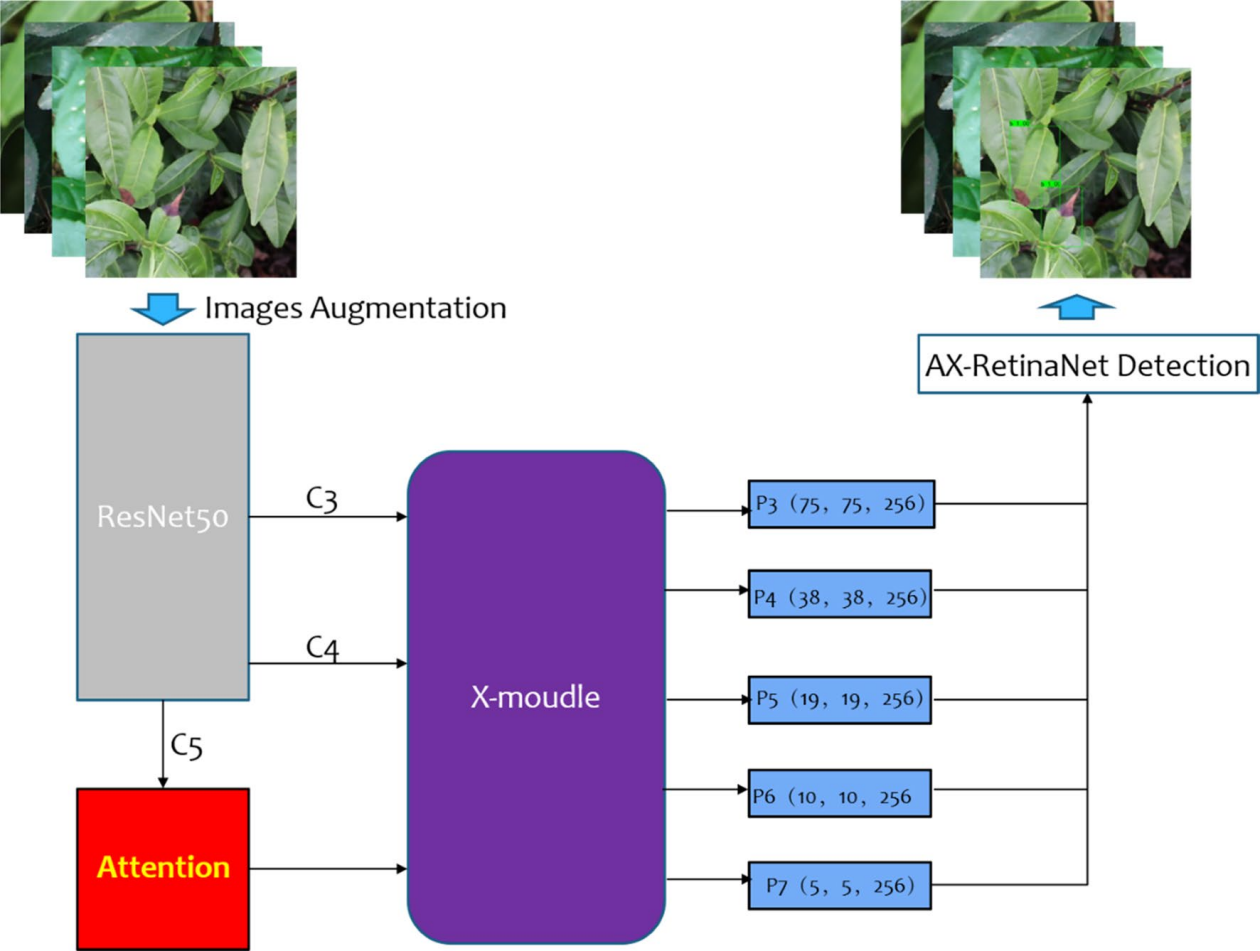
Overall framework of AX-RetinaNet.

The overall framework of AX-RetinaNet is shown in Fig. 6. AX-RetinaNet uses an improved multiscale feature fusion module X-module and adds a channel Attention module. The X module fuses the multi-scale features of the C3 and C4 layers extracted by Resnet-50 multiple times to obtain feature layers with rich semantic information, which overcomes the problem of the disappearance of the gradient of small targets in the downsampling process. In this way, the effective feature layers can be obtained through multi-scale feature fusion, thereby reducing the misdetection of the network.

**Figure 7:**
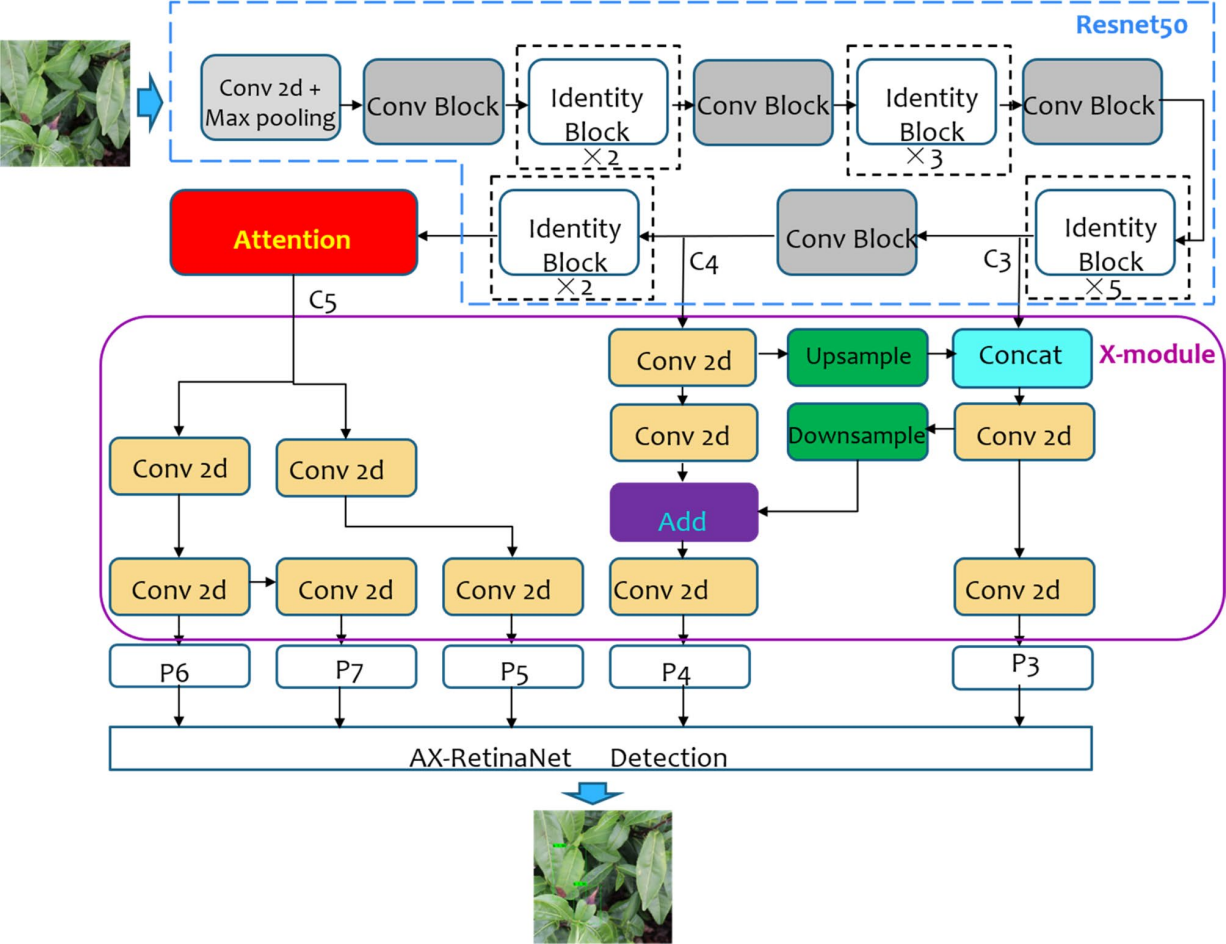
Structure of AX-RetinaNet.

AX-RetinaNet adds the Attention module to the last feature C5 layer extracted by Resnet-50 to obtain enhanced features with rich spatial information. The Attention module assigns each feature map channel to the weight optimized adaptively by the network, so that the network can focus more attention to the useful feature layers and reduce the interference of redundant features and the error detection results. The structure of AX-RetinaNet is shown in Fig. 7.

**Figure 8:**
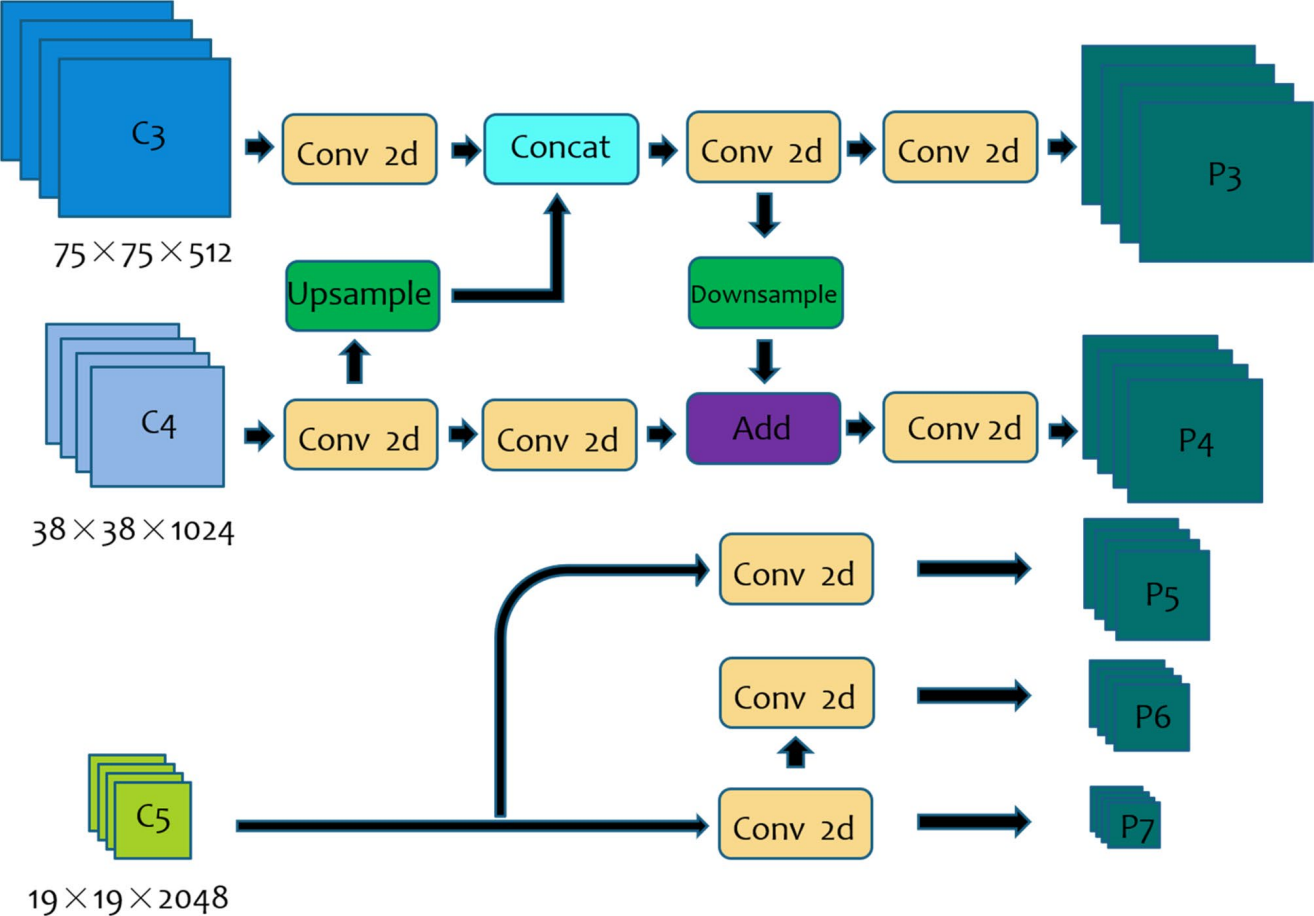
Structure of feature fusion module X-module.

The structure of the feature fusion module X-module is shown in Fig. 8. It uses the features of the lower C3 and C4 layers extracted by ResNet-50 through up-sampling and down-sampling to obtain effective feature layers P3 and P4 with rich semantic information to ensure that the classification and regression network can detect and identify targets more accurately. The high-level features with rich spatial information extracted by ResNet-50 are processed by the channel Attention module, and then the obtained features are inputted into the X-module module to obtain feature layers P5, P6, and P7. Finally, the X-module outputs the multiscale feature information of the target to solve the problem of the scale changes of the target.

**Figure 9:**
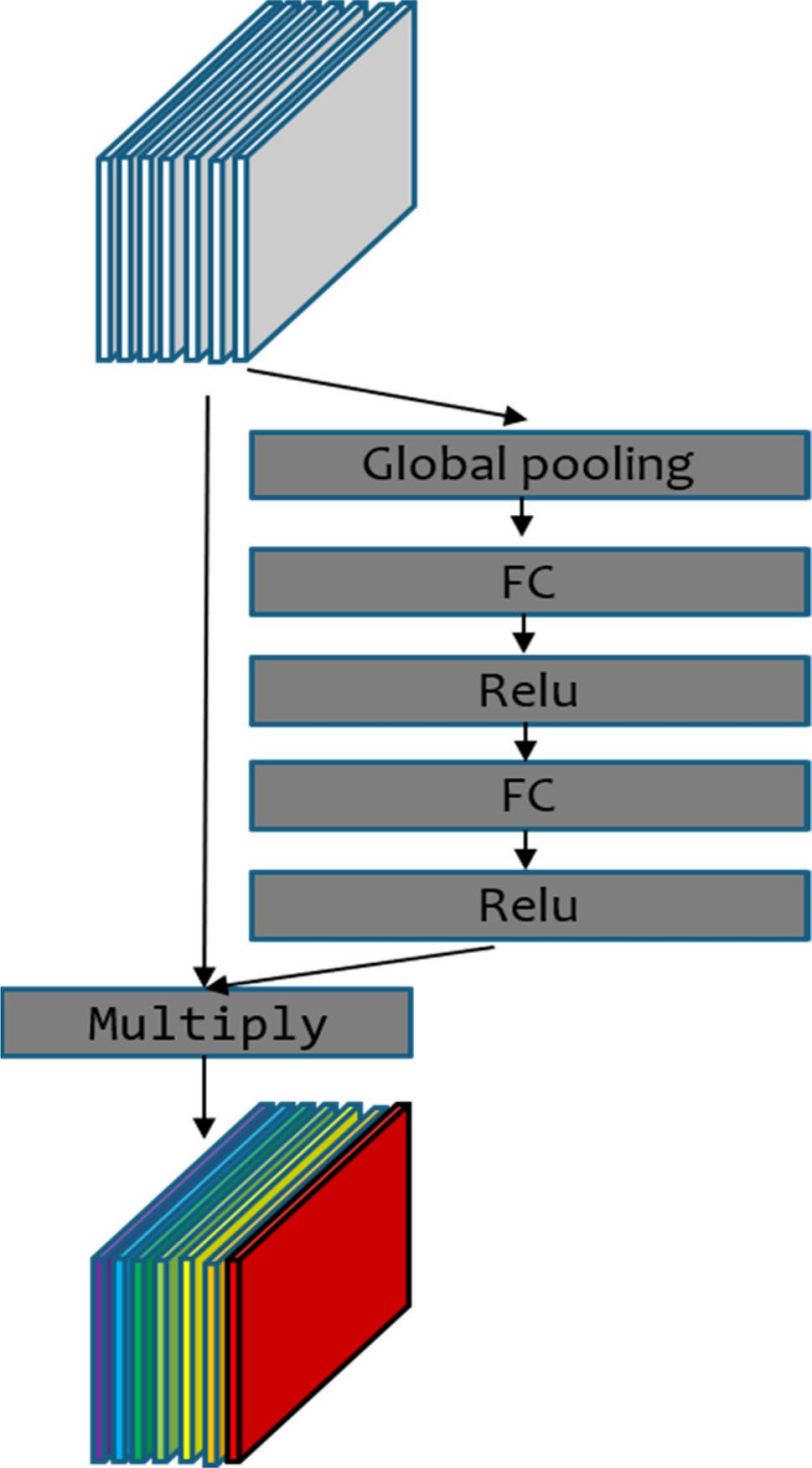
Structure of the channel Attention module.

The attention mechanism redistributes the originally evenly allocated resources according to their importance. Important resources are given greater weights, and the unimportant ones are given smaller weights. The weights are optimized adaptively according to the network structure and sample distribution. In this study, the channel Attention module is added to AX-RetinaNet, which assigns different weights to the high-level feature layer channels extracted by the backbone network Resnet-50. The structure of the Attention module is shown in Fig. 9. The inputs of the module are feature maps without adding weights, and then the feature maps are processed by the global pooling layer, the fully connected layer, and the Relu activation function to obtain the weight values of the different channels of the feature maps. Finally, the input feature maps of the module are multiplied by the weight values, and the module outputs enhanced the high-level feature maps with rich spatial information. Because these weights are optimized adaptively by the network during the training process, the channel Attention module in AX-RetinaNet can make the network pay more attention to useful features and reduce the interference of redundant information.

### Algorithm steps

First, the acquired tea images of each type of disease are divided into a training set and test set according to 4:1. The annotation tool LablImg is used to manually annotate the disease locations and disease types on the images. The images of diseased tea leaves in the training set are expanded using the proposed image augmentation method to solve the problem of insufficient training data and prevent network overfitting. All the images are adjusted to 600 × 600 size, and the augmented auxiliary images are filled with gray bars to square and adjusted to the same size. Second, the initialization parameters of AX-RetinaNet are set, and the weights of the VOC pre-training model are loaded to make the network converge faster^32^. The training images are input into the AX-RetinaNet, and the network parameters are trained and saved. Finally, the test images are input into the trained network to obtain the results of the tea leaf disease detection and identification.

The specific steps of the algorithm are as follows:

- **Step 1** The acquired tea images of each type of disease are divided into the training set and test set according to 4:1.
- **Step 2** The disease locations and disease types on the images are annotated using the annotation tool LablImg.
- **Step 3** The number of images in the training set is expanded using the image augmentation method.
- **Step 4** All the images are adjusted to 600 × 600 size, and the augmented auxiliary images are filled with gray bars to square and adjusted to the same size.
- **Step 5** The initialization parameters of AX-RetinaNet are set, and the weights of the VOC pre-training model are loaded.
- **Step 6** The training images are input into AX-RetinaNet, and the network parameters are trained and saved.
- **Step 7** The test images are input into the trained network models to obtain the results of tea leaf disease detection and identification.

## Experimental results and analysis

The experiment was executed on an NVIDIA Tesla V100 server using the Python language and the Keras learning framework. The deep networks are trained and tested by the virtual environment built by Anaconda3. VOC’s pre-training weights were used to initialize the network weights. The initial learning rate is set to 0.0001, the batch size is 4, momentum is 0.9, weight decay is 0.0005, and Adam optimization is used.

### Comparison of the AX-RetinaNet and classical CNNs

The detection and identification results of AX-RetinaNet and the classical CNNs, such as SSD, RetinaNet, YOLO-V3, YOLO-V4, Centernet, M2det, and EfficientNet are compared and analyzed to verify the performance of AX-RetinaNet. The Precision, Recall, and F1-score values of the detection and identification results of different networks without training image augmentation and after using training image augmentation are shown in Table 1. In Table 1, the Precision, Recall, and F1-score are defined as follows:

1 $$ Precision = \frac{TP}{{TP + FP}}, $$

2 $$ Recall = \frac{TP}{{TP + FN}} , $$

3 $$ F1{\text{-}}score = \frac{2 \times Precision \times Recall}{{Precision + Recall}}, $$

where TP is the number of true positives, FP is the number of false positives, TN is the number of true negatives, and FN is the number of false negatives. The Precise Recall curve (P–R curve) is obtained by taking the Precision value as the vertical axis and the Recall value as the horizontal axis. The area ratio occupied by the P–R curve is the AP value of the P–R curve, and mAP is the average value of the AP values. AP is defined as follows:

4 $$ AP = \int_{0}^{1} {P(r)\; dr,} $$

where P(r) is the P–R curve, and the mAP index balances the relationship between the Recall value and the Precision value, and better reflects the detection and identification performance of the network. The mAP values of the detection and identification results of different networks without training image augmentation and after using training image augmentation are shown in Fig. 11.

**Table 1:** Precision, Recall, and F1-score values of the detection and identification results of different networks.

Network	Augmentation	Precision (%)	Recall (%)	F1-score
SSD^33^	No	89.5	83.75	0.865
	Yes	87	90	0.885
RetinaNet	No	89.5	83.75	0.865
	Yes	95.5	90	0.927
Yolo V3	No	92	69	0.789
	Yes	75.25	91.5	0.825
Yolo V4	No	97.5	71.75	0.827
	Yes	96.75	80	0.876
Centernet^34^	No	98.75	57	0.723
	Yes	99.50	79.5	0.884
M2det	No	88.75	77.5	0.827
	Yes	93.5	86	0.896
EfficientNet^35^	No	94.5	81.25	0.873
	Yes	98.25	84.25	0.907
AX-RetinaNet	No	91.25	86.75	0.889
	Yes	96.75	94	0.954

**Figure 10:**
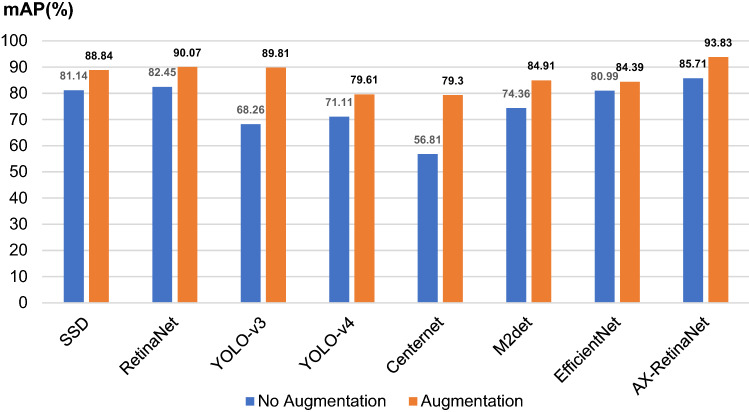
mAP values of the detection and identification results of different networks.

Table 1 and Fig. 10 show that the SSD, RetinaNet, EfficientNet, and AX-RetinaNet have good detection and identification results with or without data augmentation. After using the augmentation method, the detection and identification results of all networks are greatly improved. Among them, the performance of the Centernet improved the most. Its detection and identification results without data augmentation are as follows: the F1-score value is 0.723 and the mAP value is only 56.81%. When the augmentation method is used, its detection and identification results are as follows: the F1-score value reached 0.884 and the mAP value reached 79.30%. For EfficientNet, which had the smallest performance improvement, the mAP value of its detection and identification results increased by nearly 4%, and F1-score increased from 0.873 to 0.907, indicating that the augmentation method has a significant improvement on the detection and identification performance of the network. In addition, comparing the detection and identification results of AX-RetinaNet and the classical CNNs, the detection and identification performance of AX-RetinaNet is better than other classical CNNs. The detection and identification results of the AX-RetinaNet without data augmentation are as follows: F1-score value is 0.889, the mAP value is 85.71%, and the Recall value is 86.75%. After the training images are augmented, the detection and identification results of AX-RetinaNet are as follows: F1-score value is 0.954, mAP value is 93.83%, and Recall value is as high as 94%, which are much higher than the detection and identification results of the classical CNNs. Therefore, regardless of whether the data augmentation method is used, AX-RetinaNet achieved the highest values of mAP, F1-score, and Recall. Experimental results show the detection and identification results of AX-RetinaNet are better than those of SSD, YOLO-V4, YOLO-V3, Centernet, M2det, EfficientNet, and RetinaNet.

### AX-RetinaNet and RetinaNet performance analysis

**Figure 11:**
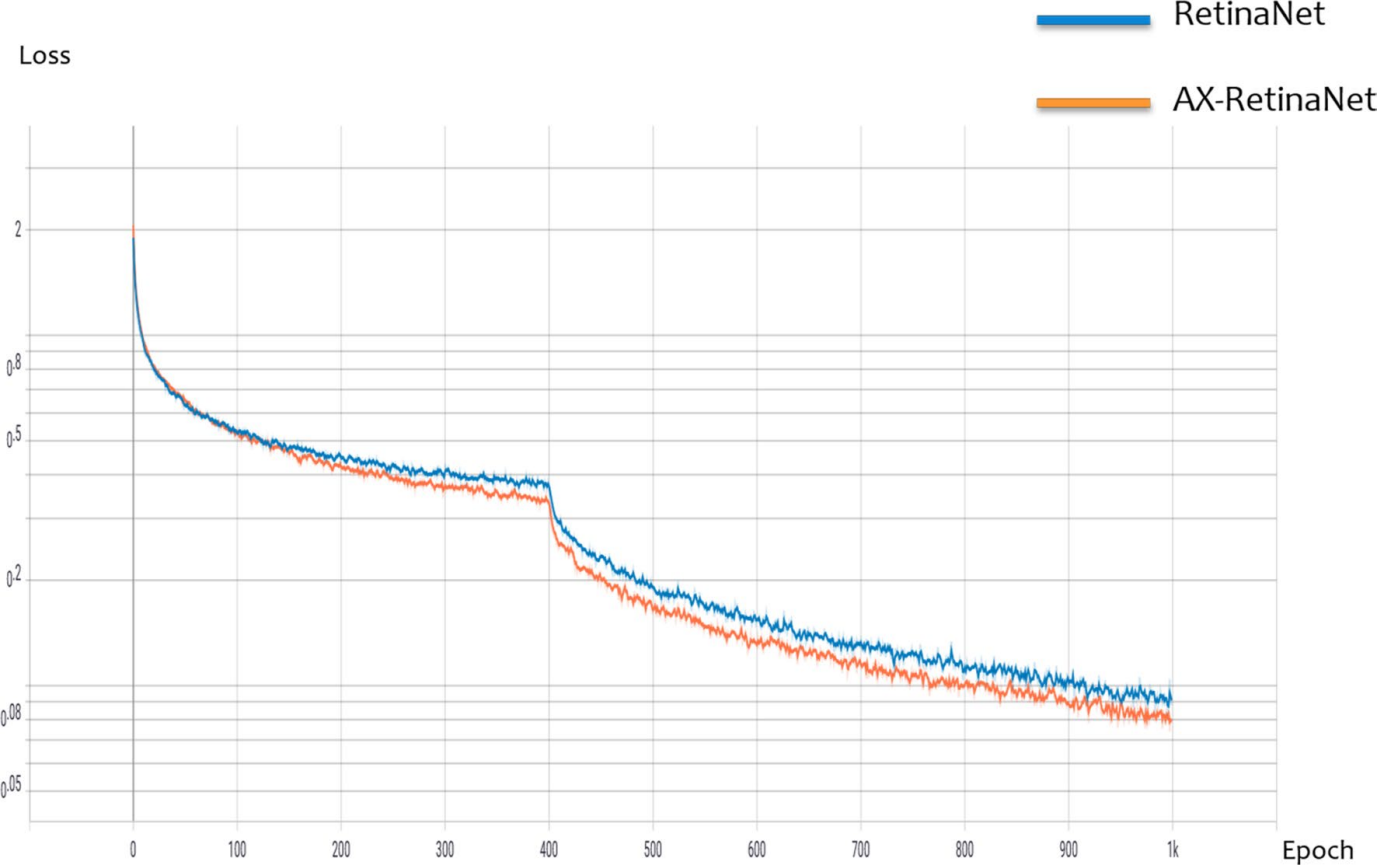
Loss curve of AX-RetinaNet and RetinaNet during training.

**Figure 12:**
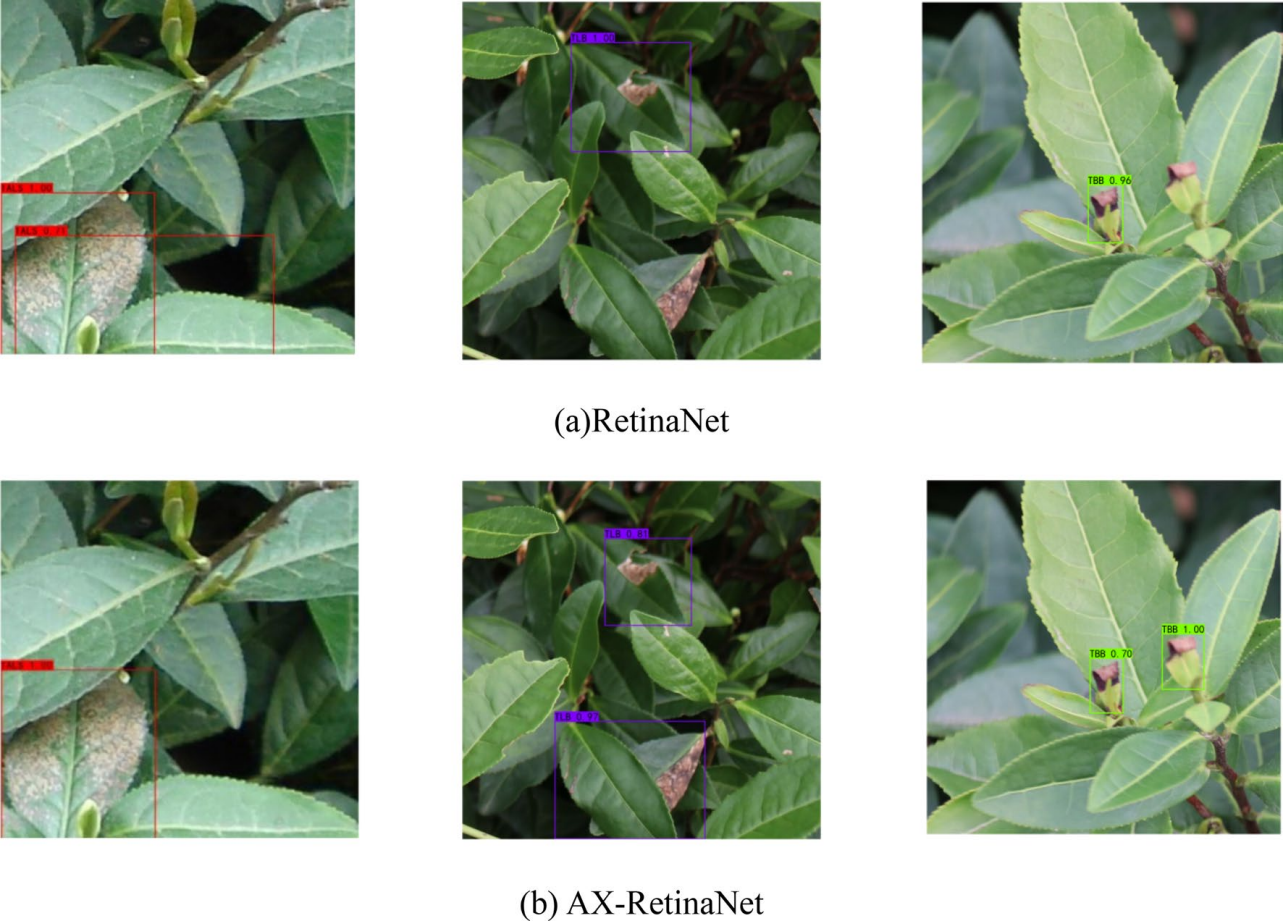
Some of the detection and identification results of AX-RetinaNet and RetinaNet.

Figure 11 shows the loss curve of RetinaNet and AX-RetinaNet during training. The figure shows that compared to RetinaNet, AX-RetinaNet reduces the loss value to a lower level and is easier to converge. Figure 12 shows some of the detection and identification results of RetinaNet and AX-RetinaNet. In Fig. 12, the red box indicates that the target is detected and identified as TALS, the purple box indicates that the target is detected and identified as TLB, and the green box indicates that the target is detected and identified as TBB. Each box is marked with the possibility of being detected and identified as a disease and the type of disease. Figure 12 shows that AX-RetinaNet can not only effectively avoid the error detection of generating multiple detection boxes on a target (left images of Fig. 12), but also reduce the misdetection of identifying diseased leaves as healthy leaves because of occlusion (middle images of Fig. 12). At the same time, it can also reduce misdetection caused by diseased leaves that have not been detected because of small objects (Right images of Fig. 12). Therefore, the improved network AX-RetinaNet reduces the error detection or misdetection of tea leaf diseases in complex backgrounds and improves the accuracy of disease identification.

### Comparison of the augmentation method used in this study and rotation augmentation method

**Figure 13:**
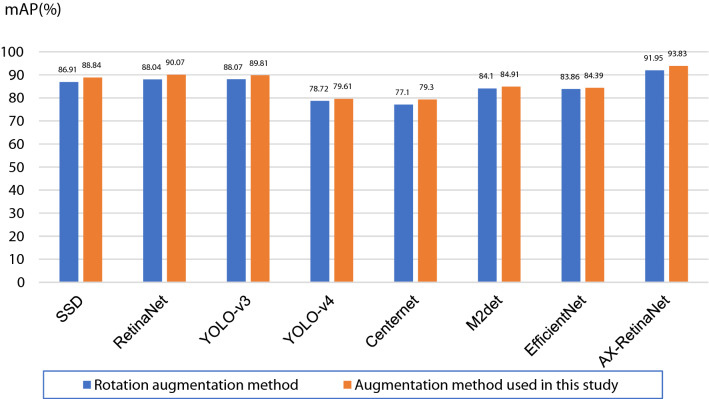
mAP values of the detection and identification results of different networks using different augmentation methods.

Rotation augmentation is used to rotate the original images by four angles (90°, 180°, 270°, and 360°) to achieve the expansion of the number of training images. The augmentation method used in this study is to add auxiliary images cut from the original images based on rotation augmentation, thereby expanding the number of training images in the training set. The Precision, Recall, and F1-score values of the detection and identification results of different networks using different augmentation methods are shown in Table 2, and the mAP values are shown in Fig. 13. The experimental results show that using the augmentation method employed in this study, AX-RetinaNet can reach an mAP value of 93.83%, which is an increase of nearly 2% compared with the rotation augmentation method, and the F1-score from 0.930 increased to 0.954. After using the augmentation method to expand the number of training images, the F1-score and mAP values of the detection and recognition results of other classical CNNs also improved to a certain extent, indicating that the image augmentation method used in this study is better than the rotation augmentation method and that it can better improve the performance of the network to detect and identify tea diseases.

**Table 2:** Precision, Recall, and F1-score values of the detection and identification results of different networks using different augmentation methods.

Network	Augmentation method	Precision (%)	Recall (%)	F1-score
SSD	Rotation	87	88.5	0.877
	Used in this study	87	90	0.885
RetinaNet	Rotation	94.75	88.5	0.915
	Used in this study	95.5	90	0.927
Yolo V3	Rotation	86	89	0.875
	Used in this study	75.25	91.5	0.825
Yolo V4	Rotation	97.75	79	0.874
	Used in this study	96.75	80	0.876
Centernet	Rotation	99	77.25	0.868
	Used in this study	99.50	79.5	0.884
M2det	Rotation	96.5	85	0.904
	Used in this study	93.5	86	0.896
EfficientNet	Rotation	97.25	84.25	0.898
	Used in this study	98.25	84.25	0.907
AX-RetinaNet	Rotation	93.75	92.25	0.930
	Used in this study	96.75	94	0.954

### Ablation experiment

AX-RetinaNet is constructed based on RetinaNet by adopting an improved feature fusion module X-module and adding a channel Attention module. This study conducted ablation experiments to analyze the impact of these two modules on detection and identification performance. The results of the ablation experiment are shown in Table 3. In Table 3, the mAP value of the detection and identification results of the original RetinaNet is only 90.07%, and the F1-score is 0.927. By replacing the FPN module in the RetinaNet with X-module, the mAP value is increased to 91.56%, and the F1-score value is increased to 0.928. The channel Attention module is added to RetinaNet, the detection and recognition effect of the network has been greatly improved, the mAP value has been increased to 92.97%, and the F1-score has also been increased to 0.935. The AX-RetinaNet is constructed by adding the channel Attention module and replacing the FPN module of the RetinaNet with the feature fusion module X-module. The results show that AX-RetinaNet can achieve the best detection and identification results in which the mAP value reached the highest at 93.83%, and the F1-score value also reached the highest at 0.954. Thus, by combining the channel Attention module and the feature fusion module X-module, the proposed network can obtain the best detection and identification results.

**Table 3:** Results of ablation experiments.

Backbone (Resnet50)	FPN	X-module	Attention	mAP (%)	F1-score
√	√			90.07	0.927
√		√		91.56	0.928
√	√		√	92.97	0.935
√		√	√	93.83	0.954

### Effects of the channel attention module on the classical CNNs

**Figure 14:**
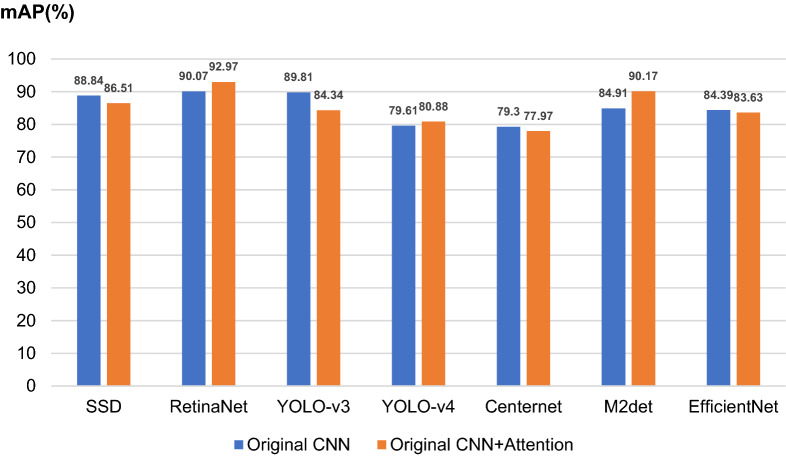
mAP values of different CNNs with or without channel Attention module.

In this experiment, the channel Attention module is added to the last layer of the backbone feature extraction network of other CNNs to further explore its effects other CNNs. The mAP values of the detection and identification results of different CNNs with or without channel Attention modules are shown in Fig. 14. The results show that the channel Attention module only improves the performance of some CNNs, such as RetinaNet, YOLO-v4, and M2det. Meanwhile, the performance of SSD, Centernet, and EfficientNet was reduced, thereby indicating that the channel Attention module does not fit with all CNNs. The reason is that the position where the channel Attention module is added to the network is very important in improving the performance of the network. Adding the channel attention module to the last layer of the backbone feature extraction network of all CNNs is not necessarily the most suitable.

The channel Attention module also plays a role in enhancing and filtering feature information. Some classical CNNs, such as RetinaNet and YOLO-V4, have deeper networks and a large number of parameters, which will extract a considerable amount of useful and redundant information. Thus, it is necessary to enhance and filter the acquired features. However, for CNNs, such as SSD and EfficientNet, their backbone networks have a small number of parameters, and the extracted feature information is insufficient. If the extracted features are further filtered, some useful features will be missed, which will affect the detection and recognition effect of the networks.

## Conclusion

Tea leaf diseases have severely affected the yield and quality of tea. Automatic detection and identification of tea leaf diseases with high accuracy is conducive to precise prevention and control of the diseases. Existing CNNs have low accuracy in detecting and identifying tea leaf diseases in natural scene images because of the problems of complex backgrounds, dense leaves, and large size changes in tea images taken in natural environments. Thus, by improving the RetinaNet, this study provides a target detection and identification network AX-RetinaNet for use in the automatic detection and identification of tea leaf diseases in natural scene images. AX-RetinaNet adopts X-module to fuse the multi-scale features of the target to obtain feature maps with rich information and adds a channel, the Attention module, to select effective features and reduce the interference of redundant features. This study also uses an image expansion method to expand the number of training images to avoid overfitting, and improve the detection and identification performance of the network. Experimental results show that AX-RetinaNet is superior to the existing classical CNNs, such as SSD, RetinaNet, YOLO-v3, YOLO-v4, Centernet, M2det, and EfficientNet, in detecting and identifying tea leaf diseases. The proposed method can be used for high-precision automatic detection and identification of tea leaf diseases in natural scene images.

## References

[1] Hu G, Yang X, Zhang Y, Wan M (2019). Identification of tea leaf diseases by using an improved deep convolutional neural network. Sustain. Comput. Inform. Syst..

[2] Zhao Y, Gong L, Huang Y, Liu C (2016). A review of key techniques of vision-based control for harvesting robot. Comput. Electron. Agric..

[3] Wang Q, Nuske S, Bergerman M, Singh S (2013). Automated crop yield estimation for apple orchards. Exp. Robot..

[4] Chaudhary A, Kolhe S, Kamal R (2016). An improved random forest classifier for multi-class classification. Inf. Process. Agric..

[5] Hossain, M. S., Mou, R. M., Hasan, M. M., Chakraborty, S. & Abdur Razzak, M. Recognition and detection of tea leaf’s diseases using support vector machine. in *Proceedings—2018 IEEE 14th International Colloquium on Signal Processing and its Application, CSPA 2018* 150–154 (Institute of Electrical and Electronics Engineers Inc., 2018). 10.1109/CSPA.2018.8368703

[6] Sun Y, Jiang Z, Zhang L, Dong W, Rao Y (2019). SLIC_SVM based leaf diseases saliency map extraction of tea plant. Comput. Electron. Agric..

[7] Hu G, Wei K, Zhang Y, Bao W, Liang D (2021). Estimation of tea leaf blight severity in natural scene images. Precis. Agric..

[8] Castelao Tetila E, Brandoli Machado B, Belete NAS, Guimaraes DA, Pistori H (2017). Identification of soybean foliar diseases using unmanned aerial vehicle images. IEEE Geosci. Remote Sens. Lett..

[9] Ayan E, Erbay H, Varçın F (2020). Crop pest classification with a genetic algorithm-based weighted ensemble of deep convolutional neural networks. Comput. Electron. Agric..

[10] Picon A (2019). Crop conditional convolutional neural networks for massive multi-crop plant disease classification over cell phone acquired images taken on real field conditions. Comput. Electron. Agric..

[11] Jiang F, Lu Y, Chen Y, Cai D, Li G (2020). Image recognition of four rice leaf diseases based on deep learning and support vector machine. Comput. Electron. Agric..

[12] Hu G, Wu H, Zhang Y, Wan M (2019). A low shot learning method for tea leaf’s disease identification. Comput. Electron. Agric..

[13] Xiong Y, Liang L, Wang L, She J, Wu M (2020). Identification of cash crop diseases using automatic image segmentation algorithm and deep learning with expanded dataset. Comput. Electron. Agric..

[14] Tetila EC (2020). Detection and classification of soybean pests using deep learning with UAV images. Comput. Electron. Agric..

[15] Krisnandi D (2019). Diseases classification for tea plant using concatenated convolution neural network. Commun. Inf. Technol..

[16] Zhao, Q. *et al. *M2Det: A single-shot object detector based on multi-level feature pyramid network. http://www.aaai.org

[17] Ren, S., He, K., Girshick, R. & Sun, J. Faster R-CNN: Towards real-time object detection with region proposal networks. arXiv:1506.01497 (2015). 10.1109/TPAMI.2016.2577031 27295650

[18] Zhang J (2020). Multi-class object detection using faster R-CNN and estimation of shaking locations for automated shake-and-catch apple harvesting. Comput. Electron. Agric..

[19] Hu G, Wang H, Zhang Y, Wan M (2021). Detection and severity analysis of tea leaf blight based on deep learning. Comput. Electr. Eng..

[20] Tiwari V, Joshi RC, Dutta MK (2021). Dense convolutional neural networks based multiclass plant disease detection and classification using leaf images. Ecol. Inform..

[21] Redmon, J., Divvala, S., Girshick, R. & Farhadi, A. You only look once: Unified, real-time object detection. arXiv:1506.02640 (2015).

[22] Redmon, J. & Farhadi, A. YOLOv3: An incremental improvement. https://pjreddie.com/yolo/

[23] Bochkovskiy, A., Wang, C.-Y. & Liao, H.-Y. M. YOLOv4: Optimal speed and accuracy of object detection. arXiv:2004.10934 (2020).

[24] Tian Y (2019). Apple detection during different growth stages in orchards using the improved YOLO-V3 model. Comput. Electron. Agric..

[25] Kang H, Chen C (2020). Fruit detection, segmentation and 3D visualisation of environments in apple orchards. Comput. Electron. Agric..

[26] Yu Y, Zhang K, Yang L, Zhang D (2019). Fruit detection for strawberry harvesting robot in non-structural environment based on Mask-RCNN. Comput. Electron. Agric..

[27] Su D, Qiao Y, Kong H, Sukkarieh S (2021). Real time detection of inter-row ryegrass in wheat farms using deep learning. Biosyst. Eng..

[28] Lin, T.-Y., Goyal, P., Girshick, R., He, K. & Dollár, P. Focal loss for dense object detection. arXiv:1708.02002 (2017). 10.1109/TPAMI.2018.2858826 30040631

[29] Gomez Selvaraj M (2020). Detection of banana plants and their major diseases through aerial images and machine learning methods: A case study in DR Congo and Republic of Benin. ISPRS J. Photogram. Remote Sens..

[30] Woo, S., Park, J., Lee, J.-Y. & Kweon, I. S. CBAM: Convolutional block attention module. arXiv:1807.06521 (2018).

[31] He, K., Zhang, X., Ren, S. & Sun, J. Deep residual learning for image recognition. in *Proceedings of the IEEE Computer Society Conference on Computer Vision and Pattern Recognition* vols. 2016-December 770–778 (IEEE Computer Society, 2016).

[32] Everingham M (2010). The PASCAL Visual Object Classes (VOC) Challenge. Int. J. Comput. Vis..

[33] Liu, W. *et al.* SSD: Single shot multibox detector. 10.1007/978-3-319-46448-0_2 (2015)

[34] Zhou, X., Wang, D. & Krähenbühl, P. Objects as points. arXiv:1904.07850 (2019).

[35] Tan, M. & Le, Q. V. EfficientNet: Rethinking model scaling for convolutional neural networks. arXiv:1905.11946 (2019).

